# HIV Drug Resistance Mutations in Proviral DNA from a Community Treatment Program

**DOI:** 10.1371/journal.pone.0117430

**Published:** 2015-01-30

**Authors:** Anne Derache, Hyoung-Shik Shin, Maya Balamane, Elizabeth White, Dennis Israelski, Jeffrey D. Klausner, Alexandra H. Freeman, David Katzenstein

**Affiliations:** 1 Stanford University, Infectious Diseases Department /Medical School, Stanford, California, United States of America; 2 Center for Infectious Diseases National Medical Center, Seoul, South Korea; 3 University of California, Los Angeles, California, United States of America; 4 Kaiser Permanente San Francisco, California, United States of America; University of Ottawa, CANADA

## Abstract

**Background:**

Drug resistance mutations archived in resting memory CD4+ cells may persist despite suppression of HIV RNA to <50 copies/ml. We sequenced pol gene from proviral DNA among viremic and suppressed patients to identify drug resistance mutations.

**Methods:**

The Peninsula AIDS Research Cohort study enrolled and followed over 2 years 120 HIV infected patients from San Mateo and San Francisco Counties. HIV-1 pol genotyping by bulk sequencing was performed on 38 DNA and RNA from viremic patients and DNA only among 82 suppressed patients at baseline. Antiretroviral susceptibility was predicted by HIVDB.stanford.edu.

**Results:**

Among 120 subjects, 81% were on antiretroviral therapy and had been treated for a median time of 7 years. Thirty-two viremic patients showed concordant RNA and DNA genotypes (84%); the discordant profiles were mainly observed in patients with low-level viremia. Among suppressed patients, 21 had drug resistance mutations in proviral DNA (26%) with potential resistance to one, two or three ARV classes in 16, 4 and 1 samples respectively.

**Conclusions:**

The high level of genotype concordance between DNA and RNA in viremic patients suggested that DNA genotyping might be used to assess drug resistance in resource-limited settings, and further investigation of extracted DNA from dried blood spots is needed. Drug resistance mutations in proviral DNA in 26% of subjects with less than 50 copies/ml pose a risk for the transmission of drug resistant virus with virologic failure, treatment interruption or decreased adherence.

## Introduction

Antiretroviral therapy (ART) has changed the dynamic of the HIV epidemic through suppression of HIV-1 RNA to undetectable plasma levels (<50 copies/ml). However, latent HIV infection of CD4+ T cells, established early after infection, necessitates lifelong ART [1–3]. Treatment failure can lead to the selection of drug resistance mutations (DRM) in the viral genome, which may be archived in the cellular reservoir. Even when viral RNA (vRNA) is suppressed, DRM may be detected in proviral DNA by sequencing extracted DNA from buffy coat, whole blood or peripheral blood mononuclear cells (PBMC) [4–7]. DRM archived in proviral DNA may be expressed upon virologic failure, transmit drug resistant virus and impact management of long-term ART [8–11]. Although it is the current standard for assessing drug susceptibility, the plasma genotype may underestimate drug resistance if treatment has been interrupted or adherence compromised with the overgrowth of susceptible virus in the absence of drug pressure [12–14].

Genotyping of the proviral reservoir may inform treatment decisions, identify occult DRM and uncover community phylogenetic relationships. Retrieving HIV resistance data from PBMC may be less expensive, but the clinical significance of DRM in proviral DNA remains controversial [9,15–17]. Moreover, consensus genotype from plasma RNA or proviral DNA does not detect DRM as minority variants <20% [18–20].

Integrated HIV proviral DNA in T central memory cells can be transcriptionally reactivated leading to virological failure [1,2,21]. Some studies show that HIV proviral populations may evolve despite apparent suppression of viremia [15], while others find minimal changes in the composition of the proviral population during suppressive treatment [22,23]. The proviral reservoir is most likely the source of the persistent expression of virus at <50 copies/ml among patients on suppressive ART, despite the addition of potent integrase inhibitors [24–26]. Whether these low levels of HIV RNA result from the continued replication in reservoirs, tissues or cells unaffected by ART, or are derived from activation of cells in the latent reservoir is controversial, and both mechanisms may be occurring.

The resistance mutations detected by genotyping proviral DNA after virologic failure have been found to closely mirror those obtained from concomitant vRNA [4,27–29] unless there has been a treatment interruption or poor adherence [30]. Significantly fewer mutations have been found in proviral DNA compared to historic vRNA genotypes showing that some DRM are not archived or are not detectable in the latent reservoir [30–32]. Conversely, the archived provirus mutations in T cells may not be detected in plasma RNA in the absence of drug pressure [33,34].

Here, to assess the prevalence and the pertinence of DRM archived in proviral DNA, 120 subjects, 82 suppressed to <50 copies/ml and 38 with 68-171,000 copies/ml of plasma RNA, were sequenced from one or both compartments. We identified virologic failures among suppressed patients by evaluating the longitudinal relationship between archival DNA and RNA drug resistance patterns. PBMC genotyping may identify transmission linkages and add information to optimize ART among suppressed patients.

## Material and Methods

### Study population

One hundred and twenty HIV infected individuals were recruited from the Peninsula AIDS Research Consortium clinic at the San Mateo County AIDS Program and the San Francisco Public Health Department clinic, and enrolled in a longitudinal study of drug resistance. Participants were recruited from public health clinics during attendance for routine HIV care.

### Ethics statements

Written informed consent was obtained for each participant and the study was approved by the institutional review board “Administrative Panels for the Protection of Human Subjects” from Stanford University for the protocol 7861 “Antiretroviral Drug Resistance in Blood, Oral, and Genital HIV-1: Implications for Pathogenesis and Transmission of Drug Resistance and Secondary Prevention in Community Based Treatment Programs”.

### Sample collection

Study visits occurred every 6 months over a total of 2 years. Blood specimens were obtained at each visit and transported to the laboratory within 6 hours of collection. Plasma was removed after centrifugation and PBMC were separated from whole blood by density gradient centrifugation and stored at -70°C in 90% human serum, 10% Dimethyl Sulfoxyde (DMSO). Viral load (VL) and CD4 measures were performed by the clinical laboratories at San Mateo County hospital using Roche Cobas Amplicor Monitor v1.5 (Roche Molecular Diagnostics, Inc., Branchburg, NJ, USA) and BD FACSCalibur (Becton Dickinson, Franklin Lakes, NJ, USA), respectively.

### DNA and RNA extractions

Virus was concentrated from 1 ml of plasma by centrifugation for 20 min at 20,000g to pellet the virus, 800μl of supernatant was discarded and RNA extracted from the 200μl residua with QIAamp viral RNA Mini kit (QIAGEN Inc., Hilden, Germany) as per the manufacturer’s protocol. vRNA was used as template for reverse transcription using SuperScript II Reverse Transcriptase (Invitrogen, Carlsbad, CA, USA) and random hexamer primers, using cycling parameters of 10min at 25°C, 30min at 45°C and 15min at 70°C, and the RNA template degraded with RNAse H for 20 min at 37°C. DNA was extracted from pelleted PBMC using QIAamp DNA Blood Mini kit (QIAGEN Inc.).

### Genotyping

At baseline, vRNA and proviral DNA, were sequenced from viremic patients (n = 38) while suppressed patients (n = 82) provided only a proviral DNA genotype. Only the vRNA was sequenced from patients who virologically failed during follow-up (n = 11).

A 1,300-bp fragment encompassing the protease (PR) and the first 300 amino acids of the reverse transcriptase (RT) gene was generated from DNA and cDNA for PCR amplification, followed by a nested PCR with 1^st^ round PCR primers; MAW26 (5´-TTGGAAATGTGGAAAGGAAGGAC-3´) and RT21 (5´-CTGTATTTCTGCTAT TAAGTCTTTTGATGGG-3´) and 2^nd^ round with PRO1 (5´-CAGAGCCAACAGCCC CACCA-3´) and RT20 (5´-CTGCCAGTTCTAGCTCTG CTTC-3´), with Platinum Taq DNA Polymerase (Invitrogen), 50pmoles of each primer, 2mM of Magnesium Chloride and 0.5mM of dNTPs. Forty cycles for the 1^st^ and 35 for the 2^nd^ round of 94°C for 15s, 55°C for 20s and 72°C for 2 min, then 10min at 72°C produced a nested PCR product, purified using QIAquick PCR Purification kit (QIAGEN Inc.), as per manufacturer’s protocol. After quantification, 30ng were sequenced using the Big Dye Terminator kit (Life Technologies, Foster City, CA, USA) and a set of 4 bidirectional primers by capillary electrophoresis on a 3130xl Genetic Analyzer (Life Technologies).

### Sequence analysis

Sequences were assembled using Geneious R6 genetic analyzer (Biomatters, Auckland, NZ) [35]. DRM were identified using the Stanford HIV Drug Resistance Database [36]. A Neighbor Joining phylogenetic tree was inferred with Phylip package [37] with 2-Kimura parameters and support values for the branching pattern were calculated from 1000 bootstrap replicates, visualized using FigTree [38] and analyzed for potential transmission clusters and linked infections. The frequency of apolipoprotein B mRNA-editing, enzyme-catalytic, polypeptide-like 3G/F (APOBEC3G/F) G to A mutations have been determined with the Calibrated Population Resistance tool from the HIV Stanford Database for Quality Assessment [39]. More than 3 characteristic mutations in the RT and 2 in the PR genes associated with APOBEC3G/F activity were considered as evidence for hypermutation. HIV-1 PR and RT sequences recovered from PBMC and viral RNA have been submitted to GenBank with the accession numbers KJ69464 to KJ769641.

## Results

### Baseline characteristics

One hundred and twenty subjects were enrolled: their demographic, immunologic and virologic baseline characteristics are presented in Table 1. The median age of the study population was 44 years (ranging from 20 to 64), 82% were male, 38% White, 28% African-American, 25% Hispanic/Latino and 9% Asian/Pacific Island/Native American (reflecting the ethnic and gender distributions of HIV infection in the San Francisco Bay Area). Among all study participants, 97 (81%) had been on ART for a median of 7 years. Of these patients, 41% remained on a non-nucleoside reverse transcriptase (RT) inhibitor (NNRTI) and 59% on a Protease inhibitor (PI) based regimen.

**Table 1:** Demographic, immunologic and virologic characteristics of the study population n = 120.

Characteristic		N = 120
Gender	Female	21 (17.5%)
	Male	98 (81.7%)
	Transgender	1 (0.8%)
Ethnicity	White	46 (8.37%)
	African American	33 (27.5%)
	Hispanic / Latino	30 (25%)
	Other (Asian, Natives)	11 (9.2%)
Age	Median (range)	44 (20–64)
On HAART	Yes	97 (81%)
CD4 count *(cells/mm3)*	<200	22 (18%)
	>200	98 (82%)
Viral Load *(copies/ml)*	<50	82 (68.3%)
	>50	38 (31.7%)
Patients on HAART	Viral Load < 50	79 (81.5%)
	Viral Load >50	18 (18.5%)
	CD4 count < 200	17 (17.5%)
	CD4 count > 200	80 (82.5%)
Patients not currently on HAART	Viral Load < 50	3 (13%)
	Viral Load >50	20 (87%)
	CD4 count < 200	5 (12%)
	CD4 count > 200	18 (78%)

At study entry 82 subjects (68%) were suppressed to <50 copies/ml and 38 (32%) had >50 copies/ml of plasma HIV-1 RNA with a median of 5,865 copies/ml. The median CD4+ cells count was 422/mm^3^ among suppressed patients and 305/mm^3^ among viremic patients. Cumulative ART history included exposure to TDF (85%), AZT and/or d4T (57%) and 57% and 66% had received an NNRTI- or PI- based regimen respectively.

### Baseline resistance profiles in viremic patients

**Fig 1:**
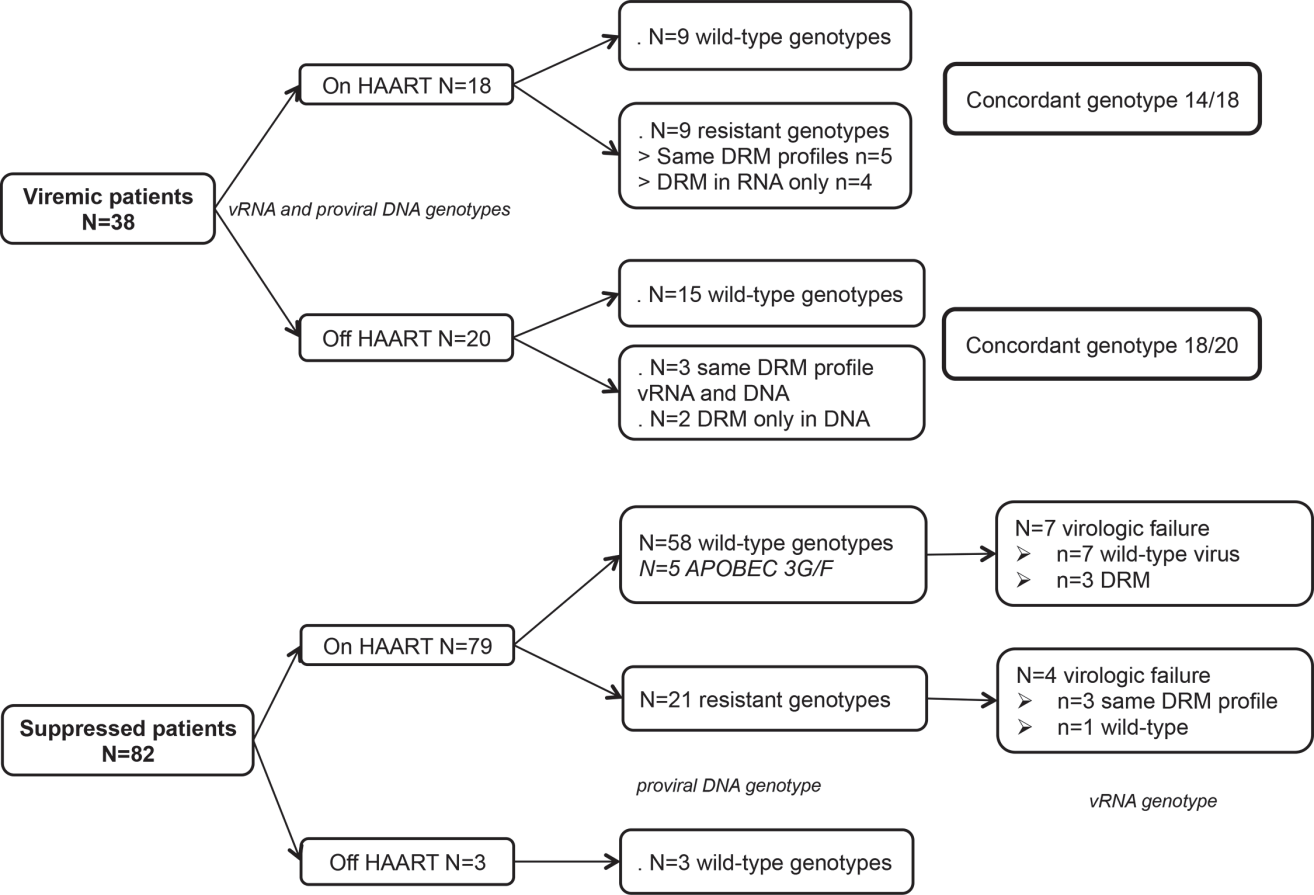
Flow chart for the 120 patients enrolled in the study.

Overall, resistance profiles in both vRNA and proviral DNA were concordant in 32/38 viremic patients at baseline (84%). Twenty-four patients had wild-type genotype in both vRNA and DNA and 14 had DRM (see Table 2); 12 had resistance mutations in vRNA and DNA with identical DRM in 8/12 (some present in vRNA were absent in proviral DNA), and 2 had DRM only in DNA (see Fig. 1 and Table 2).

**Table 2:** Drug resistance mutations profiles of 14 viremic patients at baseline with drug resistance with their viral load and treatment status at entry.

PID	ART at baseline	VL at baseline	Proviral DNA				Viral RNA			
			TAMs	Non TAMS	NNRTI	PI	TAMs	Non TAMs	NNRTI	PI
10064	yes	67			K103N			M184MV	K103N	
10020		68	T215FIST, K70KR, D67DN, K219KQ				T215F, K70R, D67N, K219Q, **M41L, T69ADNT**	M184V		
20012		149	T215Y, M41L	M184MV		D30N, M46L	T215Y, M41L, **L210W**	M184V		D30N, M46L
10041		788	T215ILPT			I54V, V82IT	T215IL			I54V, V82IT
50007		969	D67DG, K219KN	M184MV	K103KN, V106AV		D67G, K219N	M184V, **L74IL**	K103N, V106A	
10014		3,320	T215F	M184MV			T215F	M184V		
10056		7,190	T215NSTY	M184V	K103N		T215Y	M184V	K103N	
60009		41,700			K103N, Y181C				K103KN, Y181YC	
10047		49,100			V179D				V179D	
60001	no	156		M184V		V82AITV				
10046		2,770		M184MV						
30018		4,114			K103R				K103R	
30014		12,548			K103N				K103N	
30023		29,268			K103N				K103N	

The other 24 viremic patients did not have DRM in either RNA or DNA.

Discordant DRM between proviral DNA and viral RNA are shown in blue.

Among viremic patients, 18/38 (47%) had been on treatment at baseline for a median of 4 years (ranging from 1 to 13 years). Half were wild-type and the other half harbored resistance in vRNA and proviral DNA, and 4 of them displayed DRM in vRNA that were not present in proviral DNA (see Table 2 – upper section).

Twenty viremic patients were not treated or had interrupted treatment prior to enrollment, 15 of whom had wild-type genotype in both vRNA and proviral DNA. Three of the twenty untreated patients had DRM in both vRNA and DNA, and 2 had DRM only in proviral DNA (see Table 2 – lower section).

Discordant resistance profiles in vRNA and DNA were seen in 6 patients with low viral load (mean of 697 copies/ml) in contrast with the concordant genotypes among 32 with higher virus load (mean 31,252 copies/ml). Considering only viremic patients with VL >1,000 copies/ml, concordance between vRNA and proviral DNA was 97%. Finally, among 12 patients known to have been previously exposed to NNRTI, 4 had NNRTI resistance mutations in proviral DNA while among 22 exposed to PI, only 3 had PI resistance mutations.

### Baseline resistance profiles in suppressed patients

Most of the 82 suppressed subjects were on treatment, 3 were reported to be off of treatment and harbored wild-type genotypes in proviral DNA (see Fig. 1). Twenty-one harbored archived DRM in proviral DNA (26%); 11 to NRTI, 5 to NNRTI only, 3 to NNRTI and NRTI, 1 to PI and NRTI, and 1 had DRM to all the 3 classes. Three of the subjects had NNRTI resistance mutations in proviral DNA although previous NNRTI exposure had not been recorded. Among the 12 subjects exposed to NNRTI, 6 (50%) had NNRTI resistance mutations, while among 17 patients who have been exposed to a PI only 2 (12%) harbored PI resistance mutations. Among the 61 without detectable DRM in proviral DNA, 5 (8%) had ≥3 APOBEC3G/F signature mutations in *pol* gene with modestly higher CD4 cell counts (mean value 435 vs 578 CD4 cells/mm^3^).

### Emergence of viremia and virologic failure

Of the 82 study subjects with <50 copies/ml at study entry, 11/82 (13%) subsequently had a VL >50 copies/ml at a mean of 39 weeks. Seven of the 11 had virologic failure with >1,000 copies/ml (mean VL = 28,331 copies/ml), among them 3 had DRM in proviral DNA at baseline (upper section of Table 3); 1 had an NRTI mutation (PID 10069), 1 had NNRTI mutations (PID 10082) and 1 had both NRTI and NNRTI mutations (PID 10033). Two of them had the same DRM at virological failure and one had wild-type genotype. Two patients among 4 with wild-type genotype at baseline developed new DRM consistent with NNRTI based regimen (PIDs 10017 and 50002, middle section of Table 3). Among the 4 with low level VF (mean VL = 160 copies/ml) (lower section of Table 3); one patient had NRTI mutations at baseline and virologic failure (PID 10059), and another one wild-type at baseline had a K70KR mixture arose at VF (PID 10018). The 2 other patients (PIDs 10010 and 20014) had a wild-type genotype at both baseline and virological failure.

**Table 3:** Drug resistance mutations profiles among 11 patients who were suppressed at baseline (BL) and failed ART during the study follow-up.

PID	BL DRM in DNA	Week of virologic failure—VL	vRNA TAMs	vRNA Non TAMs	vRNA NNRTI	ART regimen
10033	T69ADNT, M184MV, G190AG	48–4,710				AZT 3TC TDF NVP
		72–1,670				
10069	T215D	24–1,680	T215D			ABC AZT 3TC NVP
10082	K103KN, K238KT	48–2,130			K103KN, K238KT	TDF FTC AZT ATV
10017		24–74,200				TDF FTC EFV
		48–7,610	K65KR / M184MV		K103N, Y188FLHY, V108IV, M230ML	
10075		48–32,000				TDF FTC ATV
10090		48–73,300				TDF FTC ATV
50002		24–10,300			Y188CY	TDF FTC EFV
10010		24–59				TDF FTC NVP
		96–69				
20014		48–157				NA
10018		48–119		K70KR		TDF ABC LPV
10059	M41L, T215S	48–303		M41L, T215S		TDF FTC LPV
		72–276		M41L, T215S		

### Phylogenetic analysis

**Fig 2:**
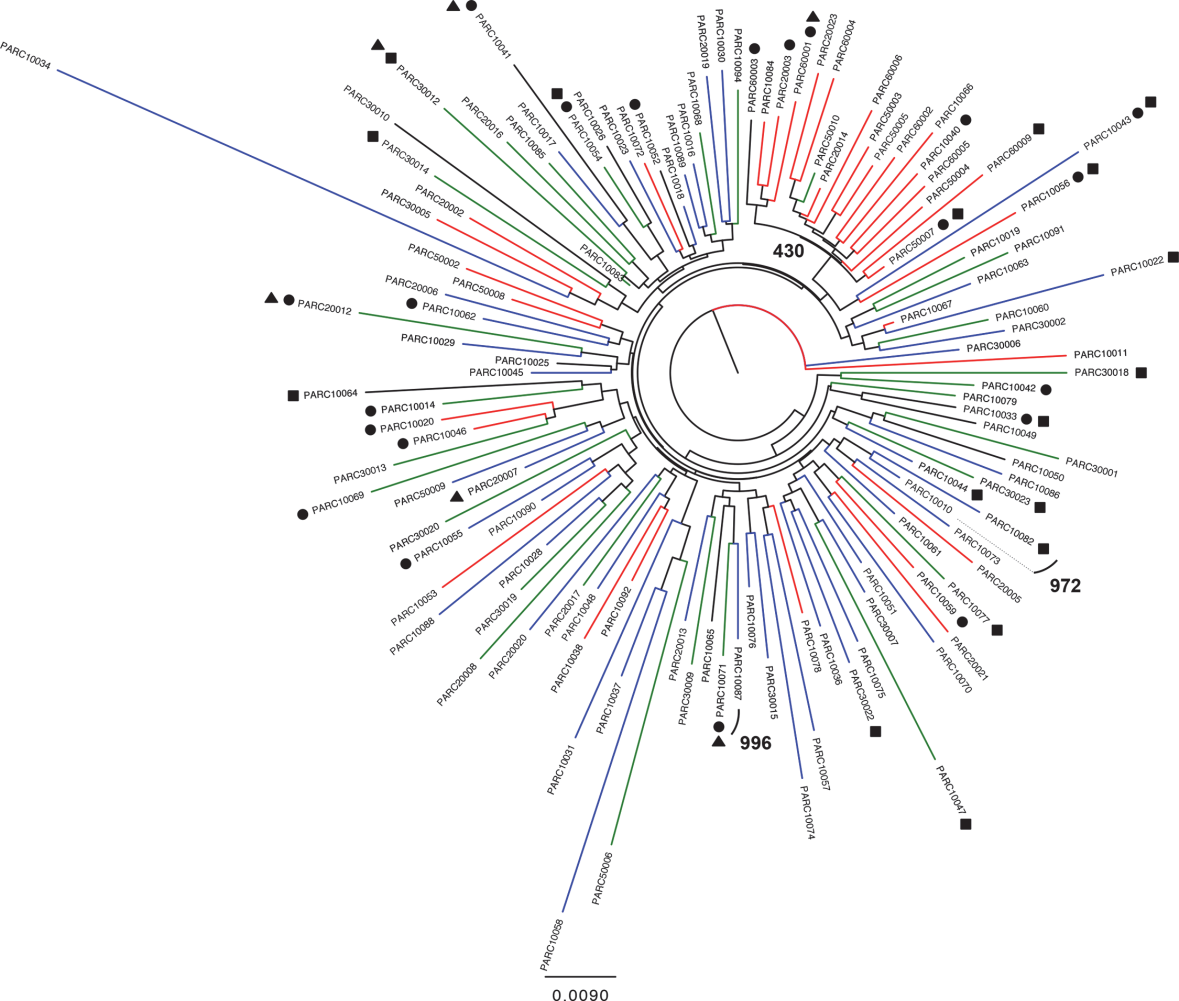
Neighbor Joining tree with 2-Kimura parameters and 1,000 replicates. Ethnicities are represent with different color codes: blue = White, green = Hispanic, red = African American and black = Asian and others. Bootstrap values for transmission clusters are showed. Drug resistance mutations are also represented ● NRTI ■ NNRTI and ▲ PI.

A phylogenetic tree shown as Fig. 2 shows 2 pairs of sequences that cluster with high bootstrap values. The first pair includes 2 men in their late 30s, one heterosexual and one a former intravenous drug user (IDU). The second pair of men in their late 30s, included a former IDU who had sex with men and a heterosexual. These risk group characteristics suggest that direct transmission was unlikely to occur, but the clustering suggests a common source of infection.

Another cluster distinguished from the tree includes 18 subjects. Their median age is 50 years, and 16 are African-American, 15 are former IDU, 10 are female and 10 currently live in East Palo Alto. Despite a low bootstrap value (430), this group shares common characteristics, which highlight the dynamic of HIV epidemic among drug users and their sexual partners in the 1990s [40].

## Discussion

In summary, our study showed that proviral DNA and vRNA drug resistance profiles of viremic subjects are concordant (84%, CI95%: 69–92%) and are consistent with a median of 87% describe in the literature [4,13,41–45]. Discordance was most common among those with low virus loads (50–1,000 copies/ml). Among those with >1,000 copies/ml, there was 97% concordance between mutations detected in viral RNA and PBMC DNA.

RNA genotyping is the gold-standard template for HIV drug resistance testing and requires RNA extraction, reverse-transcription followed by PCR and sequencing reactions. vRNA is labile and genotyping protocols mandate separation and storage of plasma at -70°C within 6 hours. In addition, reverse transcription is the most critical and costly step in RNA genotyping [46]. While this study used cryopreserved PBMC, which can be labor intensive to prepare and requires cold storage as well, DNA may be extracted from whole blood or dried blood spots, and can be transported at room temperature for several days. These results suggest that proviral DNA sequencing may be a less resource intensive means to assess drug resistance in resource-limited settings among patients failing ART. Further studies should compare genotyping of vRNA and proviral DNA extracted from dried blood spots to approximate to “real-life” conditions in RLS. Cheaper, easier and reliable proviral DNA genotyping may offer communities and clinics, distant from laboratory facilities, improved clinical management of HIV patients in decentralized treatment programs.

DRM, predominantly NRTI and NNRTI resistance mutations, were found in PBMC proviral DNA among 26% of subjects with suppressed plasma RNA levels who enrolled in a community treatment program in California. Those with PBMC DRM had failed previous ART and were subsequently suppressed on either a boosted PI or NNRTI based regimen. Stable reservoirs of NRTI and NNRTI DRM (particularly M184V and K103N) in PBMC are common at virologic failure of a first-line NNRTI based regimen [23,34,47–51]. Archived DRM in proviral DNA despite suppression of plasma RNA for years, raises concern about the potential for transmitted drug resistance (TDR). Among the viremic subjects in this study who denied previous NNRTI exposure, 2/16 (12.5%) had DRM in vRNA and proviral DNA, which is consistent with levels of TDR associated with acute infection and newly diagnosed patients in San Francisco [52]. The NNRTI and NRTI mutations observed included K103N, G190A and K238T and K65R, T215Y/F and M184V associated with rapid virologic failure on NNRTI first-line regimens as well as transmission [53–56]. The DRM observed in proviral DNA of suppressed subjects, may be expressed in vRNA in the genital track, and thus potentially may be transmitted by genital fluids [57].

The significance of low level viremia (between 50 and 1,000 copies/ml) remains controversial [58–61]. These values require confirmation, and few authorities would recommend drug switching with VL measures based on a single value between 50 and 1,000 copies/ml [62,63]. Among samples with <1,000 copies/ml, genotyping of PBMC and plasma demonstrated DRM in 5 of 9 from both components, and those with DRM in the plasma had similar DRM in PBMC DNA. Some discordance in specific mutations (present in one compartment or the other) was observed, but differences between plasma and PBMC did not identify new class resistance nor were they sufficient to alter treatment considerations. The sensitivity of detection of individual DRM in PBMC as compared to plasma was 84% (32/38). There were 2 instances at low virus load (156 and 2770 copies/ml) where DRM found in PBMC (M184V, V82F) were not expressed in plasma. This may be due to the re-emergence of wild-type virus with waning drug pressure and early virologic failure. Also, 4 samples displayed DRM in vRNA that were absent from proviral DNA that may be explained by the emergence of DRM not yet archived in proviral DNA or present but at levels undetectable by bulk sequencing <20%. Further studies may be required to examine minor viral quasispecies in proviral DNA with Next Generation Sequencing technologies.

In assigning clinical significance to DRM in PBMC, the study is limited by the potency of modern suppressive regimens with relatively small numbers with virologic failure over a mean follow-up of 46 weeks. However the frequency of DRM at entry among suppressed individuals who developed virologic failure 4/11 (36%) was similar to DRM found among those who remained suppressed, 17/68 (25%), suggesting that DRM in PBMC are not themselves a risk for virologic failure, and were only expressed in a fraction of those with later virologic failure.

APOBEC3 cytidine deaminases restrict endogenous and exogenous retroviruses by inducing G to A hypermutations in GG or GA dinucleotides context. HIV-1 counteracts this antiviral activity through Vif. Kieffer et al showed that hypermutated sequences comprise >9% of archived species in resting CD4+ cells [64]. In our study 5/81 proviral sequences were hypermutated (6%), consistent with recent studies [65,66], where E138K and M184I were the most common APOBEC3G/F associated mutations. These were clearly defined as codons that were hypermutated rather than mutations selected by 2^nd^ generation NNRTI and 3TC/FTC respectively and can be attributed to APOBEC3G/F editing [67,68].

Fourati et al demonstrated that the HIV-1 genome is often defective in PBMC after a long-term HAART [69]. In our study, subjects with APOBEC3G/F signature mutations had higher median CD4 counts than those without (578 vs 435 cells/mm^3^) reflecting a robust immune response. However, archival DRM, even in hypermutated sequences, can shape the phenotype of the circulating viral population through recombination with replication competent virus, leading to resistant strains [67]. Defective HIV-1 quasispecies in the form of multiple drug-resistant proviral DNA within cells can be rescued by superinfection with different subtype variants of HIV-1, HIV-2 and SIV [70].

Sequencing proviral DNA provided additional information about DRM, which may impact treatment decisions, particularly in the setting of low level viremia or suppression. Moreover, new information about phylogenetic relationships and the prevalence of DRM, albeit as archival mutations among suppressed individuals may identify historic transmission dynamics in a community. Here we found linkage among African American men and women in East Palo Alto, consistent with a reported peak in the IDU epidemic more than 20 years ago in this community [71]. Although the ultimate duration and clinical significance of proviral resistance is not fully defined, archival mutations often are expressed upon virologic failure, potentially contributing to TDR, and archived mutations may limit future treatment options. Further studies of proviral DNA sequences and sequence analysis in monitoring ART are warranted.
